# The Global Campaign, World Health Organization and *Lifting The Burden*: collaboration in action

**DOI:** 10.1007/s10194-011-0342-4

**Published:** 2011-04-22

**Authors:** Timothy J. Steiner, Gretchen L. Birbeck, Rigmor Jensen, Zaza Katsarava, Paolo Martelletti, Lars Jacob Stovner

**Affiliations:** 1 Department of Clinical Neuroscience, Norwegian University of Science and Technology, (NTNU), 7006 Trondheim, Norway; 2 Imperial College London, London, UK; 3 Michigan State University, East Lansing, MI USA; 4 Chikankata Health Services, Mazabuka, Zambia; 5 Danish Headache Centre, Glostrup, Denmark; 6 University of Copenhagen, Copenhagen, Denmark; 7 University of Essen, Essen, Germany; 8 Sapienza University of Rome, Rome, Italy

At the end of last year, we wrote of the achievements of *Lifting The Burden* (LTB) in its conduct of the Global Campaign against Headache [1]. In the time since, a notable event has occurred.

Earlier in that year, LTB was invited by the World Health Organization (WHO) to apply for admission into Official Relations with it. LTB duly did so and, in January 2011, the Executive Board of WHO considered and approved the application [2]. LTB joins about 190 other nongovernmental organizations (NGOs) worldwide.

Without question, this is an added marker of LTB’s considerable success in its formative years. It is the outcome of an objective and dispassionate assessment by WHO’s Executive Board not only of LTB’s track record but also of its future promise.

This reflection, however, is introspective. What does it mean for headache?

First, it is further acknowledgment by WHO that headache disorders are major causes of public ill-health. This is important: WHO is responsible for “providing leadership on global health matters, shaping the health research agenda, setting norms and standards, articulating evidence-based policy options, providing technical support to countries and monitoring and assessing health trends” [3]. We want headache to be not simply in WHO’s consciousness but among its priorities.

Official Relations are the only formal relations recognized by WHO [4]. According to its Constitution, a principal function of WHO “is to act as the directing and coordinating authority on international health work”, in support of which it “may make suitable arrangements for consultation and cooperation with NGOs in carrying out its international health work” [4]. More specifically, the objectives of WHO’s relations with NGOs “are to promote the policies, strategies and activities of WHO and, where appropriate, to collaborate with NGOs in jointly agreed activities to implement them” [5]. Official Relations are the basis of a long-term partnership in a shared cause, which here is reduction of the burden of headache worldwide. Under the terms of Official Relations, a work-plan of activities is agreed, and in LTB’s case these are key activities of the Global Campaign against Headache.

Second, it gives headache louder voice within WHO, and in the circle of WHO’s other partners. This is important, too. It is through WHO that migraine was included in the Global Burden of Disease Study 2000 [6], and again through WHO that LTB was asked to collaborate in the new Global Burden of Disease Study 2010 [7] and secured inclusion this time of tension-type headache and medication-overuse headache. Among the privileges conferred by Official Relations is entitlement to appoint a representative to participate, without right of vote, in WHO’s meetings [5].

Third, it gives headache voice in political circles [8–11]. It is not easy for NGOs to capture the attention of politicians, but this has to be done. Little can be achieved in translating the important discoveries of headache research into treatments administered to people with headache unless policy-makers create the health services that make these treatments available. It remains the case that the lives of the vast majority of people with headache in the world are untouched by the advances of the last 20 years.

For the Global Campaign, acceptance into Official Relations with WHO is a milestone. For collaborators in the Campaign’s programme of activities, it is a major encouragement and a spur to new ventures. For people with headache, it means the Campaign can work better on their behalf and is a promise of enhanced effectiveness in its efforts [1, 12, 13]. The fruits will be borne in the next years.
